# Perceived Barriers among Indonesian General Dentists in Providing Caries Preventive Care for Pediatric Patients

**DOI:** 10.1055/s-0043-1771336

**Published:** 2023-08-17

**Authors:** Safira Khairinisa, Febriana Setiawati, Risqa Rina Darwita, Diah Ayu Maharani

**Affiliations:** 1 Department of Preventive and Public Health Dentistry, Faculty of Dentistry, Universitas Indonesia, Indonesia

**Keywords:** preventive dental care, treatment barrier, dentist, Indonesia

## Abstract

**Objective** This study aimed to investigate Indonesian dentists' perceived barriers in providing caries prevention for pediatric patients.

**Materials and Methods** A total of 362 general dentists were included in this cross-sectional study. The participants were asked to complete a self-administered online questionnaire of dentist characteristics and perceived barriers in multiple domains (children, parents, dentists, and healthcare system-related barriers). The frequency of responses to items of the questionnaire was presented. The Mann–Whitney U test was used to compare perceived barriers by gender, Kruskal–Wallis by practice sector, and Spearman analysis was used to assess the correlation between perceived barrier with age, years of practice experience, weekly practice hours, percentage of pediatric patients, percentage of pediatric preventive care, and percentage of insured patients. A multivariate analysis was conducted through structural equation modeling.

**Results** The highest perceived barrier was found to be healthcare system-related, followed by parents, children, and dentists themselves. Most participants thought parents have poor knowledge of pediatric caries prevention ( *n* = 290; 80%), and dental care for young children emphasizes curative treatment over prevention ( *n* = 257; 70%). The multivariate analysis showed that dentists' practice sector and age affect perceived barriers and pediatric preventive care the most.

**Conclusion** Factors and barriers identified in this study must be the main focus of oral health programs, and dentists, as service providers, need proper training to address these barriers to optimize caries prevention in Indonesia.

## Introduction

Early childhood caries (ECC) is one of the most prevalent diseases affecting children under six years old. 1 2 In this population, during their golden period of growth and development, oral health problems can cause pain, speech difficulties, eating difficulties, decreased quality of life, and the need for orthodontic treatment in the future. 1 Indonesia has one of the highest prevalences of ECC, where 90.2% of 5-year-old children suffer from it. 2 To reduce the prevalence of caries, the promotion of preventive programs in the community and clinical settings is required.

Appropriate use of dental services is critical for both caries prevention and management. However, many studies have indicated inadequate dental care usage among various demographics. 3 4 5 6 This may be influenced by not only patients' and caregivers' attitudes and behaviors but also oral healthcare providers'. 3 7

Evidence shows that dentists sometimes do not follow evidence-based advice. 8 As healthcare providers, dentists cannot provide treatments without their patients' agreement and willingness to pay. 9 Patients' lack of knowledge and awareness will result in negative feelings and unacceptability of the treatment. 5 7 10 Most patients only seek treatment when they experience acute dental pain, so restorative treatment is emphasized more at that time. 3 11 Children's lack of coping skills and parents' acceptance make caring for them more difficult. 3 5 Dental caries is more likely in children whose caregivers lack knowledge and do not accept preventive treatment. 12

Dentists' lack of knowledge may result in a negative attitude toward dentists. Some claimed that they did not believe preventive measures were effective or important. 13 14 The shift over preventive-oriented treatment just recently made older dentists less likely to receive proper information throughout their dental school. 15 Also, dentists' high workload and lack of time sometimes make it impossible to provide preventive treatment. 15 16 17

The healthcare system should facilitate preventive dental care the most. 7 However, dentists thought they were not properly compensated for prevention. 7 13 Financial pressures and a lack of reimbursement make dentists not provide the treatment, nor do patients ask for it. 9 18 The accessibility of healthcare facilities and materials also influences the ease of treatment delivery. 10 18 19

In children, dental anxiety is often triggered by a negative dental experience and a fear of dentists. 20 Preventive treatments, such as regular examinations, oral prophylaxis, and topical fluoride applications, can reduce children's fear of the dentist, improving their view of dental care and encouraging them to use it as they age. 12

Understanding children's healthcare providers' views is crucial. 5 8 Understanding what deters dentists from practicing prevention in this population can significantly contribute to the success of dental caries prevention programs and reduce caries prevalence throughout the life course. 7 10 18 21 22 This study investigates barriers to provide preventive dental care for preschool children, as perceived by Indonesian general dentists in clinical settings.

## Materials and Methods

This cross-sectional study was conducted in November 2022 after being reviewed and approved by the Faculty of Dentistry, Universitas Indonesia's ethics committee (protocol No. 031101022).

### Sample Size and Eligibility Criteria

Using G*Power v.3.1.1 ( www.gpower.hhu.de ), a sample size of 105 dentists was determined to have a significance of 0.05 and a power of 95%. 23 An effect size of 0.25 was obtained from the association between dentists' attitudes and preventive practices in a prior study. 24 Given the possibility of selection bias, we try to collect data from participants throughout the country and make sure every sociodemographic criteria was represented. 25 To broaden the population and improve generalizability, the minimum sample size in this study was set at 350 dentists.

A nonprobability, snowball sampling, method was used to recruit general dentists nationwide with at least 1 year of work experience. Social media of each of the 34 provinces' Indonesian Dentists Association were used to recruit key persons who distributed the online questionnaire to general dentists in their working area through the organizations' WhatsApp group. This study excluded specialists and those who are currently not practicing.

### Data Collection

A self-administered online questionnaire was prepared by adapting previous studies regarding barriers to providing care for children and preventive treatment. 3 18 21 This study only included relevant items with the study objectives that underwent a cross-cultural adaptation process. 26 The questionnaire was validated through pilot testing on 42 general dentists at the 2-week interval and psychometric properties were assessed. All items in the questionnaire were valid (Pearson's product moment = *r* count > r table) and reliable (Alpha = 0.886; Corrected Item Total Correlation (CITC) > 0.3; Interclass Correlation Coefficient (ICC) = 0.888).

Participants who agreed to participate in this study completed the questionnaire using Google Forms. Before proceeding to the main part of the questionnaire, participants were explained the study's objectives and given their informed consent. Participants answered a five-point Likert scale ranging from “0” (strongly disagree) to “4” (strongly agree) in the questionnaire, which consisted of the child (5 items), dentist (5 items), parents (5 items), and healthcare system-related (5 items) dentists' perceived barrier. Sociodemographic (gender, age) and practice characteristics (practice sector, practice experience, weekly practice hour, weekly patients number, weekly pediatric patients number, weekly pediatric preventive care patients number, and percentage of insured patient) were also recorded. The number of patients collected was used to calculate percentage of pediatric patients and percentage of pediatric preventive care.

### Data Analysis

Data analysis was performed using SPSS 23 software (IBM Corp., Armonk, New York, United States). The means and standard deviation for numerical variables and the prevalence for categorical variables were examined using descriptive statistics. Mann–Whitney U test was used to compare perceived barriers by gender, Kruskal–Wallis by practice sector, and Spearman correlation analysis was used to assess the correlation between perceived barriers with age, years of practice experience, weekly practice hours, percentage of pediatric patients, percentage of pediatric preventive care, and percentage of insured patients.

Due to the lack of normality and different characteristics of variables, Partial Least Squares – Structural Equation Modelling (PLS-SEM) was conducted with smart PLS version 3.2.9 to determine the association between multiple variables to perceived barriers and the percentage of pediatric patients who receive preventive care simultaneously. 27 28 The coefficient of determination (R ^2^ ) calculates the proportion of the variance in the dependent variable predicted by the independent variable. The path analysis (β) determined the causal linkage between each dentist's characteristic to the perceived barrier (direct effect) and percentage of pediatric preventive care (indirect effect) using bootstrapping ( *p* -value <0.05). The model's fitness was assessed with a Standardized Root Mean Square Residual (SRMR) value. 27 28

## Results

This study analyzed data from 362 general dentists from 34 provinces in Indonesia. Most respondents (80.4%) were female, with an average age of 32.8 years and 6.7 years of work experience. Dentists in this study worked in a variety of settings: 126 (34.8%) worked in community health centers, 43 (11.9%) in government hospitals, 41 (11.3%) in private hospitals, 258 (71.2%) in private practices, and 107 (29.7%) in national health insurance covered clinics. Most of these dentists worked in more than one practice, with the highest proportion working solely in the private sector (38.4%). We collected self-reported data from the dentists regarding their working hours and the number of patients treated. They worked 27.5 hours per week and saw an average of 27 patients. However, on average, only five to six pediatric patients with two preventive treatments were seen each week. When compared to the total number of patients treated, 23.3% were pediatric patients, and 41% received preventive care. About 28.2% of the respondents worked in healthcare settings that provided national or private and insurance facilities, with a mean of 34.6% of treated patients utilizing these facilities.

Table 1 shows the proportions and means of the scores of barriers perceived by dentists related to multiple factors including child patients, parents, dentists, and the healthcare system. On the same scale (0-20), the highest mean scores of barriers for dentists were related to the healthcare system (10.93 ± 5.07), parents (10.83 ± 5.12), child patients (10.43 ± 5.37), and from the dentists themselves (5.55 ± 4.05). Respondents' answers on a Likert scale were recategorized into whether the respondent considered the item to be a barrier. Agree and strongly agree answers are considered as a barrier for the dentist. 5

**Table 1:** Agreement with statements describing barriers to preventive treatment in preschool children by general dentists ( *n* = 362)

Item	Mean (SD)	Not barrier	Barrier
**Child-related barrier (0–20)**	10.40 (5.37)		
1. Pediatric patients get upset easily	2.28 (1.32)	147 (41%)	215 (59%)
2. Pediatric patients cannot cope very well with dental treatment	1.88 (1.31)	217 60%)	145 (40%)
3. Pediatric patients do not like sitting in the dental chair	1.55 (1.33)	260 (72%)	102 (28%)
4. Most pediatric patients are fearful of dental treatment	2.30 (1.34)	141 (39%)	221 (61%)
5. The patient's poor oral health made preventive care irrelevant	2.39 (1.42)	140 (39%)	222 (61%)
**Dentist-related barrier (0–20)**	5.55 (4.05)		
1. Preventive dental care is not my priority	0.38 (0.89)	193 (53%)	169 (47%)
2. Preventive dentistry has low priority in the dental curriculum	0.85 (1.24)	305 (84%)	57 (16%)
3. There are no dental auxiliaries available to provide preventive care because I didn't ask for it	1.36 (1.45)	251 (70%)	111 (30%)
4. Preventive dental treatment is not profitable for dentist	1.87 (1.46)	346 (96%)	16 (4%)
5. I find pediatric dental treatment is stressful	1.08 (1.30)	294 (81%)	68 (19%)
**Parents-related barrier (0–20)**	10.83 (5.12)		
1. Parents have poor knowledge of pediatric caries prevention	2.80 (1.13)	72 (20%)	290 (80%)
2. Parents do not want the dentist to give their children preventive care	1.11 (1.31)	298 (82%)	64 (18%)
3. Parents do not see the need for primary teeth dental treatment	2.01 (1.37)	192 (53%)	170 (47%)
4. Parents are unwilling to pay for preventive care	2.22 (1.35)	179 (49%)	183 (51%)
a. Parents ignore regular dental visits without complaints	2.69 (1.26)	110 (30%)	252 (70%)
**Healthcare system-related barrier (0–20)**	10.93 (5.07)		
1. Dental insurance covers no preventive measures	2.30 (1.31)	172 (48%)	190 (52%)
2. Materials needed for preventive dentistry are not easily available	1.99 (1.54)	186 (51%)	176 (49%)
3. Payment for providing preventive care to children is inadequate	1.64 (1.39)	255 (70%)	107 (30%)
4. Dental care for young children emphasizes curative treatment rather than prevention	2.69 (1.24)	105 (30%)	257 (70%)
5. I think Indonesian dental care provides good service for young children	2.31 (1.33)	173 (48%)	189 (52%)

Abbreviation: SD, standard deviation.

Scale anchors: 0. strongly disagree; 4. strongly agree; agree and strongly agree (3 and 4) considered barrier.

More than half of the respondents considered three items in the child-related domain as a barrier. Dentists believe that pediatric patients are easily upset and fearful of dental treatments. Most of them come to dental health facilities with too severe oral conditions to receive preventive care. More than 50% of respondents considered two parent-related items to be barriers. According to dentists, the main barriers were parents with insufficient knowledge and parents who ignored dental visits when there were no complaints. In the dentist domain, all items were considered barriers by only less than 50% of dentists, indicating that the majority did not perceive the barriers as coming from themselves. Most of the barriers identified are in the domain of the healthcare system. More than 50% agreed that the main barriers in this domain were poor pediatric oral health services in Indonesia, insurance not covering preventive care, and a focus on curative care.

Tables 2 and 3 present an analysis of dentists' characteristic associated with their perceived barriers to determine the relationship and differences. The greater the value of these barriers, the more barriers dentists experience. Table 2 shows significant differences in the barriers experienced by dentists working in the government, private, and both sectors. Dentists working in the public sector face greater challenges.

**Table 2:** Comparative analysis between dentist characteristics and their perceived barrier ( *n* = 362)

Dentist characteristic ( *n* )	Child-related		Dentist-related		Parents-related		Healthcare system-related	
1.	X ± SD	p-value	X ± SD	*p* -value	X ± SD	*p* -Value	X ± SD	*p* -Value
Gender ^a^		0.164		0.813		0.066		0.986
Male (72)	13.3(6.3)		7.4 (5.5)		11.6(6.3)		13.4(6.3)	
Female (290)	12.3(6.4)		7 (4.7)		13 (6)		13.4(4.9)	
Practice sector b		0.002**		0.009**		0.000**		0.000**
Private (139)	11 (6.7)		6.2 (4.6)		11.6(6.2)		11.9(4.7)	
Public (88)	13.7 (6)		7.6(5.27)		14.8(5.6)		15.3(5)	
Both (135)	12.5(6.4)		7.8(4.8)		12.7(5.9)		13.8(5.4)	

Abbreviation: SD, standard deviation.

* ^a^ Mann–Whitney U test.

b Kruskal–Wallis test.

*Significant at 0.05.

**Significant at 0.01.

**Table 3:** Correlation analysis between dentist characteristics and their perceived barrier ( *n* = 362)

Dentist characteristic ( *n* )	Child-related		Dentist-related		Parents-related		Healthcare system-related	
1.	*r* -Value a	*p* -Value	*r* -Value a	*p* -Value	*r* -Value a	*p* -Value	*r* -Value a	*p* -value
Age (32.8 ± 8.2 years old)	–0.181**	0.001	–0.093	0.079	0.153**	0.004	–0.095	0.071
Practice experience (6.7 ± 6.5 years)	–0.163**	0.002	–0.045	0.396	–0.133	0.012	–0.058	0.275
Weekly practice hour (27.5 ± 16 hours)	0.172**	0.001	0.073	0.164	0.073	0.163	0.094	0.075
% pediatric patient (23.3 ± 16.7%)	–0.119*	0.023	–0.080	0.129	0.024	0.649	–0.058	0.270
% pediatric patients given preventive care (41 ± 37.2%)	–0.165**	0.002	–0.256**	<0.001	–0.245	<0.001	–0.178**	0.001
% insured patient (34.6 ± 36.3%)	0.129*	0.014	0.050	0.341	0.086	0.100	0.161**	0.002

a Spearman correlation.

*Significant at 0.05.

**Significant at 0.01.

Table 3 demonstrated the relationship between age, years of practice experience, weekly practice hours, percentage of pediatric patients, percentage of pediatric preventive care, and percentage of insured patients with the barriers faced by dentists in each domain. Practice hours in 1 week had a significant positive correlation with barriers related to the children. The higher percentage of pediatric patients showed lower dentists' perceived barriers from the children and themselves, but greater perceived barriers regarding the parents. The lower the dentists' perceived barriers in providing pediatric preventive treatment, the higher the percentage of pediatric patients treated preventively by these dentists. Dentists treating more insured patients face greater challenges in preventive measures.

**Fig. 1:**
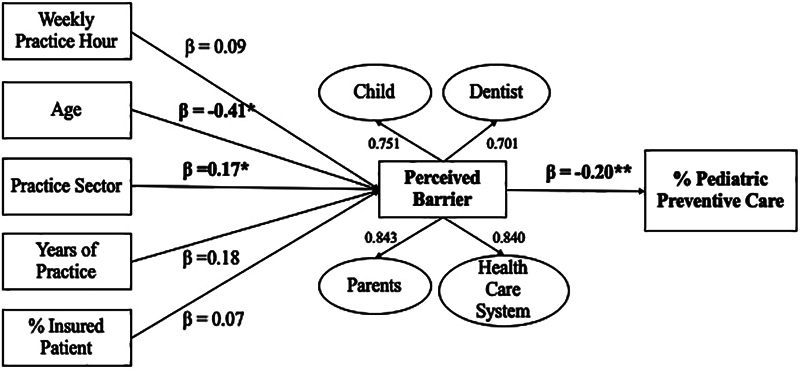
A path model showing the association between dentist characteristics with perceived barrier and percentage of pediatric patients given preventive care. Outer model = factor loading value of exogenous variables; Inner model = path coefficient value (β) of endogenous variables. *significant at 0.05; **significant at 0.01.

Fig. 1 shows the multivariate analysis to assess the association between multiple dentist characteristics to their perceived barriers and the percentage of pediatric patients given preventive care. When comparing different indicators of dentist characteristics, dentists' age (18.1%) and practice sector (16.3%) significantly contributed to fewer perceived barriers they experienced. Also, dentists' perceived barriers were associated with less percentage of pediatric patients who received preventive care significantly by 20%. All dentists' characteristics can predict perceived barriers by 12.3%, but only 4% for the percentage of pediatric preventive care provided. The relationships are shown in Table 4 . The SRMR for the imputed model was 0.051, which is under an acceptable benchmark of less than 0.08, indicating this model has a good fit. 27

**Table 4:** Direct and indirect effects of variables of dentists' perceived barrier and % pediatric preventive care among Indonesian dentists ( *n* = 362)

Variable	Direct effect (perceived barrier)		Indirect effect (%pediatric preventive care)	
	Path coefficient ( *β* ) a	*t* -statistics b	Path coefficient ( *β* ) a	*t* -statistics b
Age	–0.41	3.77*	0.08	2.90*
Practice hour	0.09	1.85	–0.02	1.57
Practice sector	0.17	3.30*	–0.04	2.54*
Years of practice	0.18	1.71	–0.04	1.57
% insured patient	0.07	1.28	–0.01	1.20
Perceived barrier (direct effect)	–		–0.20	4.65**
R ^2^ c	0.123		0.04	
SRMR d	0.051			

Abbreviation: SRMR, Standardized Root Mean Square Residual.

a The path coefficient shows the magnitude of the direct and indirect effects of exogenous variables on endogenous variables.

b The *t* -value measures whether exogenous variables have a significant effect on endogenous variables (significant at *t* > 1.68 and *p* < 0.05).

c The coefficient of determination (R ^2^ ) shows how much of the total variance of the construct is explained by the model.

e The model fit test assesses how good the model under study is (model fit if SRMR <0.08). *significant at 0.05; **significant at 0.01.

## Discussion

Dentists play a critical role in promoting positive attitudes toward oral healthcare. Health promotion and prevention are more effective than treatments alone in the long run. 10 But, in terms of providing this treatment to pediatric patients, dentists have a more challenging role. 3 12 24 Lee (2014) demonstrated that dentists believe children are irritable and that treating pediatric patients is stressful. Some also claimed that the pay for treating pediatric patients was insufficient. 3 9 Also, preventive care has many barriers from the dentist's perspective, such as a lack of knowledge, improper remuneration, and inadequate healthcare facilities. 7 18 22 This study combines these two points to investigate the barriers dentists perceive when providing preventive care to pediatric patients.

According to the findings of this study, the healthcare system was the most difficult barrier for dentists to overcome when providing preventive care. Many dentists believe that the current healthcare system prioritizes cure over prevention. Furthermore, most insurance policies not covering preventive dental care make it more difficult for dentists to provide these services. This could be due to patients' low willingness to pay, especially if they did not experience acute pain. 6 9 Thus, it is less desirable to receive preventive care without insurance. The majority of dentists, however, different from what was shown in other studies, believe that the payment received for preventive care is adequate. 3 11 18 Preventive agents that are often unavailable also make providing the treatment impossible. 29

Parents are responsible for their children and undoubtedly play a significant role in determining whether they will be treated. 5 In this study, dentists felt parents lacked knowledge about caries prevention measures and tended to skip routine dental visits. 30 Some dentists also believed that parents thought dental care was unnecessary, so dental care utilization may still be low. 4

Children's lack of coping abilities makes the treatment even harder. 3 Most of the time, primary preventive practices were no longer relevant because the oral conditions of pediatric patients who visited the dentist were too severe. 1 11 Dentists also believed that most pediatric patients feared dental treatment, making it difficult for them to provide care. 3 4 Lastly, there are barriers from the dentists themselves where some of them still think preventive dental care is less important than other treatments. 3 21 Most dentists deny that preventive dentistry is not prioritized in their dental curriculum, preventive dental care is not profitable, and pediatric dental care is stressful for dentists. However, the findings of this study contradict several previous studies that found general dentists frequently found treating children stressful. 5 Recent shifting of academic curriculum toward a greater focus on prevention may have caused these more positive perceptions due to more younger participants in this study. 31

This study also examined how dentist background affects barriers perceived by them. Female dentists may be more motivated to deliver preventive treatment and feel fewer perceived barriers. This could be since female health workers are found to be more interested in patient-centered treatment and methods. 15 Despite the unequal number of female and male dentists in this study, Indonesia has more female dentists and previous studies also showed gender disparities with similar proportions. 32

The practice sector also influences the barriers they face every day. 33 Dentists working in the government sector face more challenges, which may be related to a lack of resources, differences in clinical orientation, and the expectations of patients seeking oral care. 34 The public or primary care sector demonstrates that healthcare workers typically receive a fixed salary from the government. In contrast, private sector employees are paid on a fee-for-service basis. 35 Private healthcare is generally concerned with increasing profits, whereas the public sector is less so. 33 However, greater expenses, over-incentivization of procedures and testing, a higher risk of consequences, and looser regulation can impair private sector efficiency. The public sector is less patient-friendly and often lacks preventive care resources. 3 Healthcare spatial analysis showed that almost half of Indonesian dentists work in the private sector only, which aligns with this study's representativeness. 36

In this study, younger dentists faced more children and parents-related barriers. However, the more recent curriculum emphasizes preventive care, communication skills, and community dentistry. Therefore, new graduates should more interested in preventive approaches. 15 Inexperience may have contributed to this study's discrepancies. Also, dentists who worked greater hours felt more difficulties. Dentists know that longer working hours may be associated with stress and burnout, resulting in less-than-optimal treatment. 37

Fee-for-service is still the most common payment mechanism in developing countries like Indonesia. Thus, few can afford regular dental care, mainly only for curative purposes. Dental health treatments are underutilized because preventive measures are considered unimportant. 38 Although there are many types of private insurance in this country, Indonesian national health insurance, which is available to everyone, only provides limited coverage of preventive education and consultation. 30 Patients may decline to undergo preventative care if they must spend more money on it. 6 No matter how much dentists wish to give preventive treatment, it is impossible if their patients and parents refuse to receive it and do not use healthcare facilities effectively.

Based on the current study's findings, we suggest allocating more time and resources to promote preventive behaviors. Five Ottawa Charter principles should be prioritized—building supportive environments, constructing solid public policy, bolstering community action, empowering one's abilities, and reorienting health services. 39 In Indonesia, oral health is already considered a component of overall health in national policy. Several preventive programs, such as affordable fluoridated toothpaste, oral health education, and parental counseling, are already available. 4 Other preventive measures, such as topical fluoride, sealants, and silver diamine fluoride, are well-known and employed in various settings. Nonetheless, these approaches have not been made available nationwide due to limited resources. As the emphasis shifts from curative to preventive care, national insurance should cover more preventive measures, such as regular fluoride and sealant applications in community or clinical settings.

Cross-sectional studies have various limitations that should be considered when interpreting the results. It is impossible to say whether the variables being studied cause the outcome or whether the outcome causes the variables. Due to respondents' responses at a certain time, working hours and patient numbers may be inaccurate. Thus, recall or social desirability bias can occur. 25 Multivariate analysis may have low determinant coefficients since this study did not investigate many other variables that affect the number of patients who received treatment. Also, the non-probability sampling may result in only dentists who are knowledgeable about preventive care agreeing to participate, leading to less accurate conclusions that cannot be generalized to the entire population.

## Conclusion

This study identified multiple barriers, with the healthcare system and parents related factors posing the greatest perceived barriers. These parties must be targeted in health programs. As service providers, dentists must be trained to address these barriers (through formal or continuing education) to maximize caries prevention practices in Indonesia. Despite the economic and geographic barriers to reducing the prevalence of ECC, more research on effective strategies and actions to address this problem should be conducted. Advocating for the importance of young children's oral health is critical because it has a long-term impact on their growth and development.
